# ICTV Virus Taxonomy Profile: *Papillomaviridae*

**DOI:** 10.1099/jgv.0.001105

**Published:** 2018-06-21

**Authors:** Koenraad Van Doorslaer, Zigui Chen, Hans-Ulrich Bernard, Paul K. S. Chan, Rob DeSalle, Joakim Dillner, Ola Forslund, Takeshi Haga, Alison A. McBride, Luisa L. Villa, Robert D. Burk, ICTV Report Consortium

**Affiliations:** ^1^​ School of Animal and Comparative Biomedical Sciences, Cancer Biology Graduate Interdisciplinary Program, Genetics Graduate Interdisciplinary Program, BIO5 Institute, and the University of Arizona Cancer Center, University of Arizona, Tucson, AZ, USA; ^2^​ Department of Microbiology, The Chinese University of Hong Kong, Hong Kong SAR, PR China; ^3^​ Department of Molecular Biology and Biochemistry, School of Biological Sciences, University of California Irvine, Irvine, CA, USA; ^4^​ Sackler Institute for Comparative Genomics, American Museum of Natural History, Central Park West and 79th St., New York, NY, USA; ^5^​ International HPV Reference Center, Department of Laboratory Medicine, Karolinska Institutet, 14186 Stockholm, Sweden; ^6^​ Department of Medical Microbiology, Laboratory Medicine, Lund University, Sölvegatan 23, Sjukhusområdet, 221 85 Lund, Sweden; ^7^​ Division of Infection Control and Disease Prevention, Department of Veterinary Medical Science, The University of Tokyo, Tokyo, Japan; ^8^​ Laboratory of Viral Diseases, National Institute of Allergy and Infectious Diseases, National Institutes of Health, Bethesda, MD, USA; ^9^​ Hospital das Clínicas da Faculdade de Medicina da Universidade de São Paulo, Instituto do Câncer do Estado de São Paulo, Centro de Investigação Translacional em Oncologia, Universidade de São Paulo, São Paulo, SP, Brazil; ^10^​ Departments of Epidemiology and Population Health, Pediatrics, Microbiology and Immunology, and Obstetrics and Gynecology and Women’s Health, Albert Einstein College of Medicine, Bronx, NY, USA

**Keywords:** *Papillomaviridae*, ICTV Report, taxonomy

## Abstract

The *Papillomaviridae* is a family of small, non-enveloped viruses with double-stranded DNA genomes of 5 748 to 8 607 bp. Their classification is based on pairwise nucleotide sequence identity across the L1 open reading frame. Members of the *Papillomaviridae* primarily infect mucosal and keratinised epithelia, and have been isolated from fish, reptiles, birds and mammals. Despite a long co-evolutionary history with their hosts, some papillomaviruses are pathogens of their natural host species. This is a summary of the International Committee on Taxonomy of Viruses (ICTV) Report on the taxonomy of the *Papillomaviridae*, which is available at http://www.ictv.global/report/papillomaviridae.

## Virion

**Fig. 1.**
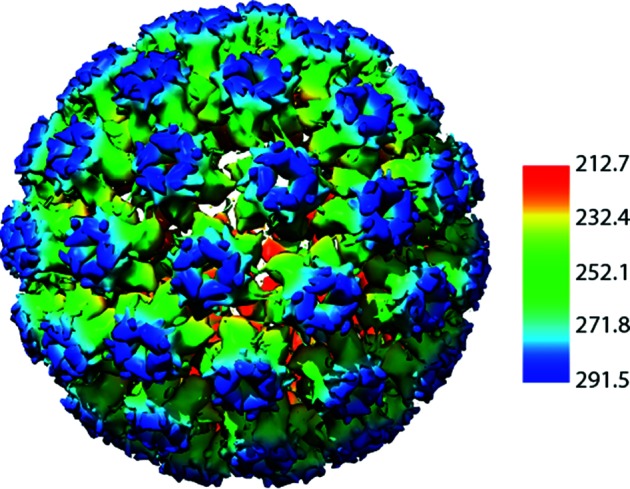
Atomic rendering of a papillomavirus capsid. Derived from an image reconstruction from cryo-electron microscopy of human papillomavirus type 16 at 4.5 Å resolution and colored according to the radial coloring scheme shown (PDB: 5KEP; [7]).

The non-enveloped viral capsid is ~600 Å in diameter and consists of 72 pentamers of the major capsid protein, L1, and ~12 molecules of the L2 minor capsid protein (Table 1, Fig. 1) [1].

**Table 1.** Characteristics of the family *Papillomaviridae*

Typical member:	human papillomavirus 16 (K02718), species *Alphapapillomavirus 9,* genus *Alphapapillomavirus,* subfamily *Firstpapillomavirinae*
Virion	Non-enveloped, 55 nm, icosahedral
Genome	Circular dsDNA genome of 5 748 to 8 607 bp
Replication	Bidirectional (theta) replication
Translation	Early and late transcripts, alternative splicing, alternative open reading frames
Host Range	Mammals, birds, reptiles and fish
Taxonomy	Two subfamilies include >50 genera and >130 species

## Genome

**Fig. 2.**
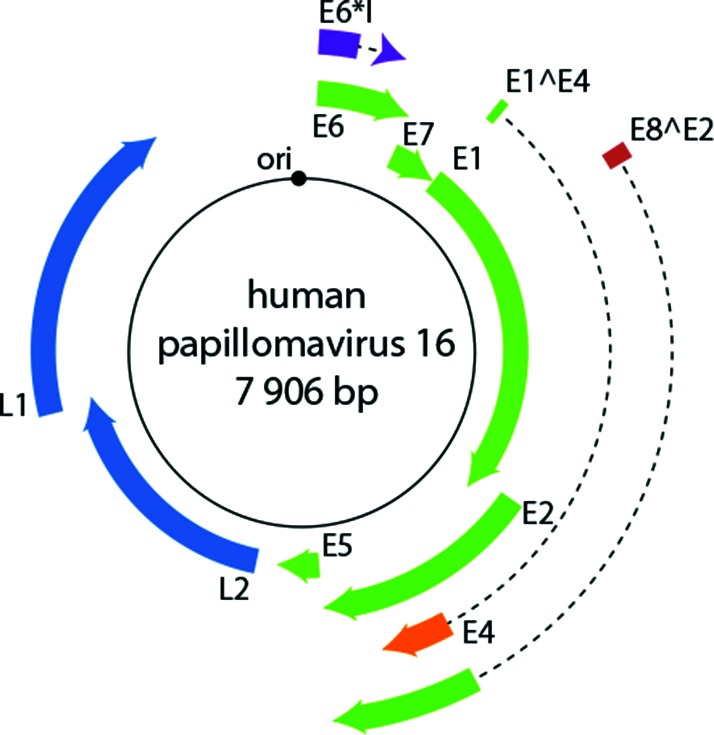
Diagram of the human papillomavirus 16 genome. The viral dsDNA is indicated. The outer boxes indicate the protein-coding open reading frames. Dotted lines represent intron sequences. The black circle represents the viral origin of replication (ori).

The viral genome varies from 5 748 to 8 607 bp. The genome comprises three functional regions. The early region encodes proteins involved in transcription, replication, and manipulation of the cellular milieu. The late region encodes the capsid proteins L1 and L2. The upstream regulatory region, located between the L1 and E6 open reading frames, contains the origin of replication as well as binding sites for viral and cellular transcription factors (Fig. 2) [2].

## Replication

Papillomaviruses primarily infect epithelial cells. Following a micro-abrasion, the incoming virion complexes with extracellular heparin sulfate proteoglycans on the basement membrane. This interaction results in conformational changes in the L1 and L2 capsid proteins, in turn allowing for transfer of the virion to an unknown entry receptor. Following furin cleavage of L2, the virion becomes internalised using a process that shares similarities with macropinocytosis. The L2-DNA complex traffics to the trans-Golgi network, until mitosis. By metaphase, the viral DNA can be seen to be associated with host chromosomes [3]. The viral replication cycle consists of three distinct phases of replication. Initial limited viral DNA amplification is supported by the viral E1 and E2 replication proteins. This initial burst of replication is followed by maintenance replication, during which the viral genome is maintained at a relatively low, but constant copy number in the proliferating cells of a lesion. Finally, as an infected cell completes cellular differentiation there is a switch towards differentiation-dependent genome amplification, and eventual generation of progeny virions [4]. During maintenance replication, the viral E6 and E7 proteins are able to usurp the cellular environment, allowing for viral replication in differentiated cells [5]. In the top layers of the differentiated epithelia, viral DNA is amplified to a high copy number and the capsid proteins self-assemble into particles encapsidating the viral DNA. As the cells slough off into the environment, infectious virions are released, completing the viral replication cycle.

## Taxonomy

Classification of the *Papillomaviridae* is based on sequence identity across the L1 open reading frame [6]. The family includes two subfamilies, *Firstpapillomavirinae*, which includes >50 genera and >130 species, and *Secondpapillomavirinae*, with a single genus and species. Genera are named according to the Greek alphabet (e.g. *Alphapapillomavirus*), with the prefixes ‘*Dyo*-’ and ‘*Treis*-’ indicating additional cycles through the alphabet (i.e. Greek for ‘a second or third time’).

## Resources

- Full ICTV Online (10th) Report: http://www.ictv.global/report/papillomaviridae.
- The Papillomavirus Episteme (PaVE): http://pave.niaid.nih.gov.
- The human papillomavirus reference center: http://www.hpvcenter.se.
- The animal papillomavirus reference center: http://www.animalpv.org.
